# Editors’ Book Table

**Published:** 1874-07

**Authors:** 


### Editors' Book Table.

NOTE. — All works reviewed in the columns of the CHICAGO MEDICAL JOURNAL may be found in the extensive stock of W. B. KEEN, COOKE & CO., whose catalogue of Medical Books will be sent to any address upon request.

BOOKS RECEIVED.

*A Treatise on Pharmacy, designed as a Text-Book for the Student, and as a Guide for the Physician and Pharmacist, etc., etc.* By EDWARD PARRISH, late Professor of Theory and Practice of Pharmacy in the Philadelphia College of Pharmacy, etc., etc. Fourth edition, enlarged and thoroughly revised by THOMAS WIGAND, Graduate of the Philadelphia College of Pharmacy; with two hundred and eighty Illustrations. Philadelphia: Henry C. Lea. 1874.

Of Dr. Parrish's great work on pharmacy it only remains to be said, that the editor has accomplished his work so well as to maintain, in this fourth edition, the high standard of excellence which it had attained in previous editions under the editorship of its accomplished author. This has not been accomplished without much labor and many additions and improvements, involving changes in the arrangement of the several parts of the work and the addition of much new matter. With the modifications thus effected it constitutes, as now presented, a compendium of the science and art indispensable to the pharmacist, and of the utmost value to every practitioner of medicine desirous of familiarizing himself with the pharmaceutical preparation of the articles which he prescribes for his patients. Books such as this have their intrinsic value to the student, but they possess a further relative value inasmuch as they fill up the vacuities in American medical literature, and render him to that extent independent of the researches of investigators in the old world generally so difficult of access.

H.

*History of the American Ambulance, established in Paris during the Siege of* 1870–71, *together with the details of its Methods and its Work.* By THOMAS EVANS, M.D., D.D.S., Ph.D., President of the American International Sanitary Committee, Commander of the Legion of Honor, Grand-Croix of the Order of St. Stanislaus of Russia, etc., etc., Author, etc., etc. London: printed for the author at the Chiswick Press, and published by Sampson, Low, Marston, Low & Searle, Crown Buildings, Fleet Street. 1873.

To the American reader, not the least interesting episode in the history of the great struggle, the Franco-German war and the eventful siege of Paris, is the conspicuous and brilliant position taken by his countrymen in mitigating the horrors of war. Nowhere since its origin, has the red cross of Geneva and its noble motto, "*Hostes dum vulnerati fratres*" been more proudly worn than by the brave little band of—better than patriots—philanthropists, whose methods and work are so admirably described in the volume before us.

The work comprises "an account of the formation of the American International Sanitary Committee of Paris, together with the History of the American Ambulance;" Report "on the organization of the American Ambulance," and on the organization of Army Hospitals," from the time of Herodotus, Dodorus Siculus, and the earlier Greek writers, down to the present time, with historical sketches of their gradual development in different countries and successive ages. The preparation of this portion of the report is evidently the work of an accomplished scholar, and a laborious and pains-taking investigator, as demonstrated by the numerous citations, from a vast array of authorities, covering a field of research both wide and varied. The relative advantages of tents and tent-barracks and permanent hospitals for the care of the sick and wounded, are elaborately discussed, and much, and just, emphasis placed upon the paramount importance of fresh air. The practicability and advantages of removing the sick and wounded is examined. The discussion of these questions is embedded, so to speak, in a mass of classical and historical lore, which remains, a monument to the scholarship and learning of the author.

In the third part, the special organization of the American Ambulance Corps is detailed, together with valuable information regarding the actual condition of Paris during the siege.

Next follow, in numerical order, a valuable report of surgical cases treated—some of the cases being illustrated photographically—the medical history of the ambulance, and a series of admirable lithographic plates.

The whole work is worthy of the subject of which it treats, and that subject, in the eyes of the philanthropist, the brightest scene of that dark and bloody drama, the Franco-Prussian war.

H.

*Lectures on the Diseases of Infancy and Childhood.* By CHARLES WEST, M.D., Fellow of the Royal College of Physicians; Physician to the Hospital for Sick Children. Fifth American from the sixth revised and enlarged English edition. Philadelphia: Henry C. Lea. 1874.

A digest of the data furnished by the observation of between thirty and forty thousand cases of sick children during a period of twenty-six years by so competent a physician as Dr. West, cannot fail to be a valuable addition to medical literature. That such is the opinion of a large proportion of the profession, the demand for the preparation of a fifth edition clearly demonstrates.

The volume comprises forty-four lectures delivered to the pupils of the Middlesex Hospital.

The author manifests his appreciation of the influences of modern civilization in establishing predispositions, by devoting sixteen of forty-four lectures to the consideration of the diseases of the brain and nervous system. And the views therein announced are in the main up to the times, although marked by a spirit of what might be termed ultra conservatism, an unwillingness to wander out of sight of ancient landmarks more especially in the field of therapeutics; while the pathological details are not quite so satisfactorily elaborated as the author's abundant opportunities for observation might have permitted. This is especially apparent in relation to epilepsy, trismus, and chorea; in regard to the latter, the author's treatment is characterized by a degree of caution which amounts to timidity, and which, while it may be justified by a strict comparison of results, is nevertheless discouraging to the tyro in neuropathology. Hope has been the incentive to every advance in knowledge; without it scientific investigation would be a dreary pursuit, soon abandoned if ever commenced.

In discussing the diseases of the respiratory system, the author becomes more confident, but even here the author's conscientiousness overshadows his boldness, and he is careful to keep well within the limits of therapeutic probabilities.

The subject of cardiac diseases is ably summarized in one chapter. The diseases of the digestive system are treated in the succeeding eight chapters in which we are glad to see, amongst other good advice, a denunciation of the "barbarous empiricism" of lancing the gums of infants "irrespective of the nature of the affection from which the child suffers," or simply to expedite a natural process. We should have been glad to hear Dr. West's opinion of the value of strychnia in some forms of stomatitis in which its effects have been so admirable in other hands; effects to be anticipated inductively, from a comparison of the pathology of the disease and the therapeutic action of the drug.

There can be little doubt that avoidance of the mischievous practice of feeding children upon "arrowroot and other farinaceous food," "which the digestive powers are quite unable to assimilate," and which is thus emphatically condemned by the author, would tend to diminish greatly the ratio of infant mortality. Moreover, we could wish that the whole profession would read appreciatively the language, and imitate the example of this expert, when he speaks of "discarding, therefore, all those preparations of arrowroot, flour, or biscuit powder, in which the vulgar repose such confidence"—in utter defiance of physiology.

The author's commendation of vaccination is couched in the following very moderate tone, "although we should take a comparatively low estimate of value of the vaccination, and confess to the fullest extent the failure in its *complete* preservation, we shall yet find in the modifying and mitigating influence which it exerts over small-pox, more than enough to make us value it as a priceless boon"—which is being very thankful for very little.

Our appreciation of the value of vaccination is quite as high as that of the author—we too regard it as a priceless boon, and base our regard upon the complete demonstration of its efficiency to eradicate small-pox, if properly used.

Taking the book as a whole there is much to commend, and nothing to condemn absolutely; its defects are rather relative, and appertain to what we have already termed a spirit of ultra conservatism. To the practitioner it will prove a thoroughly safe guide, and a repertory of extensive and varied clinical experience.

Of the typographical and general mechanical work it need only be said that, it is fully up to the high standard common to all the publications of Henry C. Lea. H.

*A Practical Treatise on the Surgery of the Genito-Urinary Organs, including Syphilis. Designed as a Manual for Students and Practitioners, with Engravings and Cases.* By W. H. Van Buren, A.M., M.D., Professor of Principles of Surgery, with diseases of the Genito-Urinary System and Clinical Surgery, in Bellevue Hospital Medical College, etc., etc.; and E. L. Keyes, A.M., M.D., Professor of Dermatology in Bellevue Hospital Medical College, etc., etc. New York: Appleton & Co. 1874.

*Observations on the Pathology and Treatment of Cholera, the result of forty years' experience.* By Jno. Murray, M.D., Inspector General of Hospital, late of Bengal. New York: George P. Putnam's Sons. 1874.

PAMPHLETS RECEIVED.

*The Mutual Relations of Physicians and Druggists.* An Address before the Graduating Class of the Massachusetts College of Pharmacy, April 22, 1874. By Charles E. Buckingham, M.D., Professor of Midwifery and Medical Jurisprudence in the Medical Department of Harvard University. Reprinted from the Boston Medical and Surgical Journal, May 7, 1874. Boston: D. Clapp & Son.

*Recerche ed Experimenti sulla natura e genesi del Miasma Palustre exposte in parte al Congresso Medico-Internazionale di Firenze.* Dal Dr. Pietro Balestra. Roma dalla Typografia Romana, Piazza Poli, No. 11. 1869.

*Syphilitic Membranoid Occlusion of the Rima Glottidis.* By Louis Elsberg, M.D., Professor of Laryngology and Diseases of the Throat in the University of New York. Reprinted from the American Journal of Syphilography and Dermatology, Jan., 1874. New York: F. W. Christern, No. 77 University Place. 1874.

*Retention of Urine.* By Alexander W. Stein, Attending Surgeon to the Charity Hospital, Professor of Visceral Anatomy and Physiology in the New College of Dentistry, etc., etc. Reprinted from the New York Medical Journal, May, 1874. New York: D. Appleton & Co., 549, 551 Broadway.

*School Hygiene.* A paper read by Dr. R. J. O'Sullivan, before the New York Academy of Medicine, June 19, 1873.

*On Intra-Uterine Fibroids.* By J. Marion Sims, M.D. D. Appleton & Co., New York. 1874.

*Urethrotomy, External and Internal Combined, in cases of multiple and difficult Stricture; with remarks on the Urethral Calibre.* By Fessenden N. Otis, M.D., Clinical Professor of Venereal Diseases in the College of Physicians and Surgeons, New York.

*The Union University Catalogue,* 1873–4. From the Albany Medical College.

*Catalogue of the Officers and Students of the University of Virginia.* Fiftieth Session, 1873–4.

*Thirty-First Annual Announcement of the St. Louis Medical College.* Winter Session, 1872–3; catalogue, 1871–2.

*Twenty-Eighth Annual Announcement of Starling Medical College,* Columbus, Ohio.

*The Twenty-Fifth Annual Announcement of the Woman's Medical College,* Philadelphia.

*The Treatment of Uterine Flexion.* By Ely Van De Warker, M.D., Syracuse, N. Y. From *Buffalo Med. and Surg. Jour.*, April, 1874.

*Observations in Electro-Therapeutics.* By A. D. Rockwell, M.D., of New York, Electro-Therapeutist to the New York State Women's Hospital. From *Buffalo Med. and Surg. Journal*, April, 1874.

*The Electrolytic Treatment of Cancer.* By A. D. ROCKWELL, M.D., etc., etc. Read before the Medical Library and Journal Association of New York.

*What Effect does Syphilis have upon the Duration of Life?* By FRED'K R. STURGIS, M.D., Asst. Surg. Manhattan Eye and Ear Hospital, New York City.

JOURNALS RECEIVED.

L'Anatomie et de la Physiologie, Journal de Paris—Mai et Juin.
The American Practitioner—June.
" Atlanta Medical and Surgical Journal—June.
" Archives of Electrology and Neurology—May—No. 1, vol. 1.
" Boston Journal of Chemistry—June.
" Boston Medical and Surgical Journal—May and June.
" Buffalo Medical and Surgical Journal—May.
" Clinic, Cincinnati—May 16, 23, 30, June 6, 13.
" City Record, New York—May 12.
" Cincinnati Lancet and Observer—June.
" Cincinnati Medical News—June.
" Canada Medical and Surgical Journal—June.
" Detroit Review of Medicine and Pharmacy—June.
" Dental Cosmos—June.
" Druggists' Circular—June.
" Kansas City Medical Journal—May, June.
" London Lancet—May, 1874.
" Medical Herald, Leavenworth, Kansas—June 1.
" Medical Times, Philadelphia—May 16, 23, 30, June 6, 13.
" Medical Record, New York—May 15, June 1.
" Medical Review, Indianapolis—May.
" Medical and Surgical Reporter, Philadelphia—May 23, 30, June 6, 13.
" Missouri Clinical Record—May, June.
" Medical News and Library—June.
" Medical Examiner—June 1.
" New York Medical Journal—June.
" Nashville Journal of Med. and Surgery—May.
" Ohio Med. and Surgical Reporter, Cleveland—May.
" Pharmacist—June.
Le Progres Medical—Nos. 16, 17, 18, 19, 20.
The Physician and Pharmacist—May.
" Pacific Medical and Surgical Journal—May, June.
" Practitioner, London—May, 1874.
" Peninsular Journal—June.
" Southern Medical Record—May.
" United States Medical and Surgical Journal—April.
" Virginia Medical Monthly—April, June.

***Annual Prize*** *of the "American Journal of Obstetrics and Diseases of Women and Children."*

Dr. B. F. DAWSON, the founder and late editor of the above Journal, offers the following Prize for the best Essay on the subjoined subject:

One hundred and fifty dollars, in gold, for the best Essay on "Congenital Deformities, and Diseases Depending on Maladies of the Uterus or Membranes."

The competing Essays must be sent to the publishers (WM. WOOD & Co., 27 Great Jones Street, New York) of the Journal on or before April 15th, 1875.

The names of the authors must accompany the manuscripts in sealed envelopes, as usual with prize papers.

The Essays may be written in the English, French, or German language; and that one to which the prize may be awarded by the censors, whose names will accompany and vouch for the verdict, is claimed for first publication in the Journal.

The subject for the second year will be announced in the Journal for May, 1875.

NEW YORK, May, 1874.

---

CLINTON, Ia., May 24, 1874.

The Clinton Co. (Ia.) Medical Society suspended from membership Drs. G. E. Wetherall, and A. H. Smith, for putting in bids and taking contracts for doing the medical business for the township poor, by the year, the Society having a by-law forbidding it, and they also considering it unfair and unjust towards a helpless class, and that it was undignified and unprofessional.

P. J. FARNSWORTH.

